# Metabolomics Study of Serum Samples of β-YAC Transgenic Mice Treated with Tenofovir Disoproxil Fumarate

**DOI:** 10.3390/ijms232415750

**Published:** 2022-12-12

**Authors:** Sindhia Kumari, Faisal Khan, Amna Jabbar Siddiqui, Nurmeen Adil, Jalal Uddin, Mufarreh Asmari, Syed Ghulam Musharraf

**Affiliations:** 1 H.E.J. Research Institute of Chemistry, International Center for Chemical and Biological Sciences, University of Karachi, Karachi 75270, Pakistan; 2 Dr. Panjwani Center for Molecular Medicine and Drug Research, International Center for Chemical and Biological Sciences, University of Karachi, Karachi 75270, Pakistan; 3 Department of Pharmaceutical Chemistry, College of Pharmacy, King Khalid University, Abha 61421, Asir, Saudi Arabia; 4 The Affiliated T.C.M Hospital of Southwest Medical University, Luzhou 646099, China

**Keywords:** tenofovir disoproxil fumarate, β-thalassemia, β-YAC transgenic mice, fetal hemoglobin induction, pharmacometabolomics, GC-MS

## Abstract

β-thalassemia is one of the most common monogenic disorders and a life-threatening health issue in children. A cost-effective and safe therapeutic approach to treat this disease is to reactivate the γ-globin gene for fetal hemoglobin (HbF) production that has been silenced during infancy. Hydroxyurea (HU) is the only FDA approved HbF inducer. However, its cytotoxicity and inability to respond significantly in all patients pose a need for an HbF inducer with better efficacy. The study describes the serum metabolic alteration in β-YAC transgenic mice treated with Tenofovir disoproxil fumarate (TDF) (*n* = 5), a newly identified HbF inducer, and compared to the mice groups treated with HU (*n* = 5) and untreated control (*n* = 5) using gas chromatography-mass spectrometry. Various univariate and multivariate statistical analyses were performed to identify discriminant metabolites that altered the biological pathways encompassing galactose metabolism, lactose degradation, and inositol. Furthermore, the decreased concentrations of L-fucose and geraniol in TDF-treated mice help in recovering towards normal, decreasing oxidative stress even much better than the HU-treated mice. The proposed study suggested that TDF can reduce the deficiency of blood required for β-thalassemia and can be used for the preclinical study at phase I/II for fetal hemoglobin production.

## 1. Introduction

β-thalassemia is a genetic disorder [1], characterized by the deficiency of β-globin chains and excess α-globin, with consequent red cell membrane damage and rapid apoptosis of early erythroblasts developing in the bone marrow [2]. Children who suffer from β-thalassemia have limited options for their treatment. One of the strategies to treat this disease is to reactivate the γ-globin gene that is already present in humans but is silenced during infant development (6–8 months’ birth age). As a result of γ-globin gene activation, the body starts to produce fetal hemoglobin which somehow reduces the blood deficiency in the body [3,4]. Different pharmacological agents have been tested at various stages of clinical trials for their ability to produce fetal hemoglobin [5]. Hydroxyurea is the only Food and Drug Administration (FDA) approved HbF inducer [6] used for sickle cell anemia disease. The treatment of β-thalassemia with HU seemed to be significantly beneficial and is used as a key therapeutic agent but with the disadvantage of cytotoxicity and response which varies from patient to patient [7,8]. Subsequently, there is a necessity to find out a better therapeutic agent for HbF induction.

Tenofovir disoproxil fumarate (TDF) is a nucleotide analogue of adenosine and functions as a reverse transcriptase inhibitor [9]. It is the FDA approved drug used for the treatment of hepatitis B infection (HBV) and human immunodeficiency virus (HIV). TDF has recently been reported as a pharmacologically active compound for HbF induction [10] as it enhanced γ-globin gene mRNA transcription, erythroid differentiation, and HbF expression in K562 cells. It has been reported that the 200 mg/kg/day dosage of TDF showed better results than HU in an in vivo study on the percentage of HbF-positive RBCs in the absence of cytotoxic and myelotoxic effects [10].

This paper describes the preclinical study of TDF in β-YAC transgenic mice by analyzing their serum metabolomics and its comparison with HU-treated samples and untreated control saline group. Metabolomics is the most diverse, authoritative, and powerful tool for monitoring altered pathways. It is capable of providing a scenario of metabolic changes that occurred in different diseases [11]. Pharmacometabolomics is, comparatively, a new branch of metabolomics and is useful to study the efficacy of drugs as well as helping in understanding the mechanism of action and identifying biomarkers associated with the organism’s response to drug treatment [12]. To the best of our knowledge, this is the first report of a pharmacometabolomics study demonstrating TDF as an HbF inducer.

## 2. Results

Low molecular weight metabolites were extracted from a total of 15 samples using standard protocol [13] which includes untreated saline water control group (*n* = 5), treatment of β-thalassemia mice with TDF (*n* = 5), and treatment of β-thalassemia mice with HU (*n* = 5). The samples were analyzed using GC-MS after using a standard derivatizing protocol [13].

### 2.1. Metabolic Profiling and Identification

**Figure 1:**
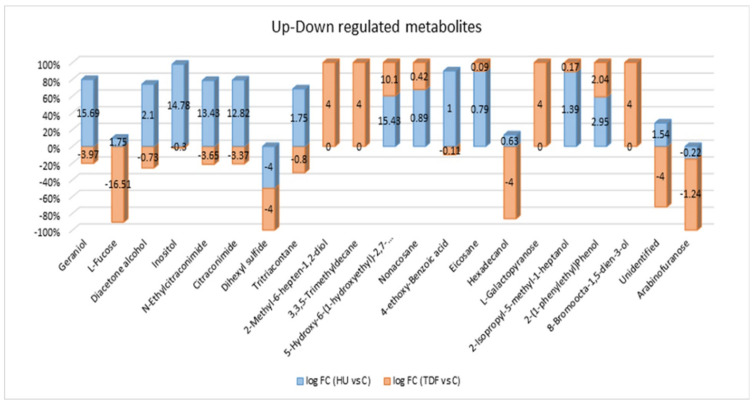
Significant metabolites showing up-regulation and down-regulation at log FC > 1.0 in two groups: blue indicates HU vs. C and red indicates TDF vs. C.

Mass Profiler Professional Software was used to generate metabolic features by importing data in CEF. file format. A total of 1345 features were extracted from all samples using different parameters. The discriminant features were extracted with the help of VIP plot of OPLS-DA. A total of 21 features were extracted showing up/down-regulation at log fold change >1.0 shown in Figure 1. The peaks of 20 entities were identified with the help of Agilent Mass Hunter Qualitative Analysis software and an already available NIST mass spectral library with match score more than 700 and similarity index ≥70%, while one was not identified at the defined similarity index. All the identified compounds were mentioned with their names whereas the unidentified were mentioned with the base peak and retention time with log FC > 1.0, VIP > 1.3, and *p* < 0.05 in Table 1. Out of 21 metabolites, 06 metabolites show up-regulation in HbF-induced TDF treatment and 08 metabolites show down-regulation in comparison with untreated control whereas others did not show any significant fold change regarding HbF-induced TDF and untreated control β-YAC transgenic mice.

### 2.2. Chemometric Analysis

**Figure 2:**
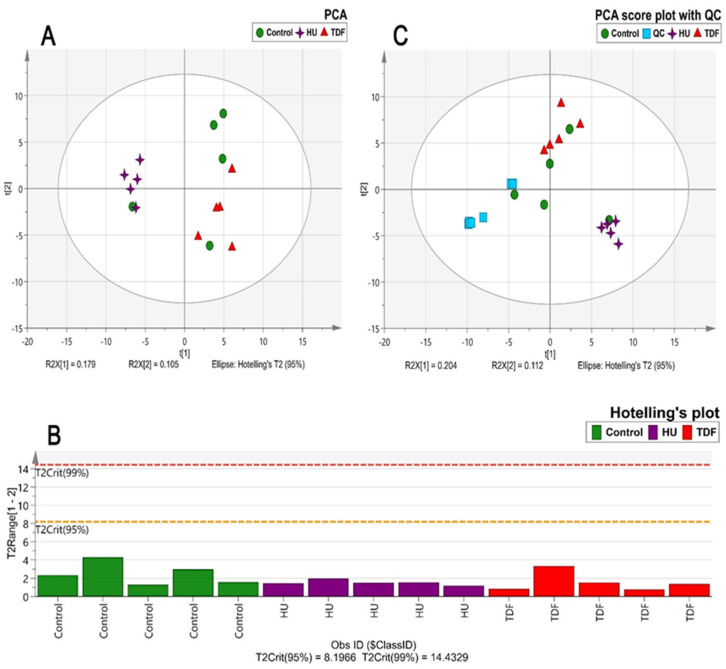
Multivariate statistical models (**A**) PCA score plot showing studied groups, (**B**) Hotelling’s T2 plot with 95% and 99% confidence limit for the outlier assessment in all groups, and (**C**) PCA score plot with QC samples.

**Figure 3:**
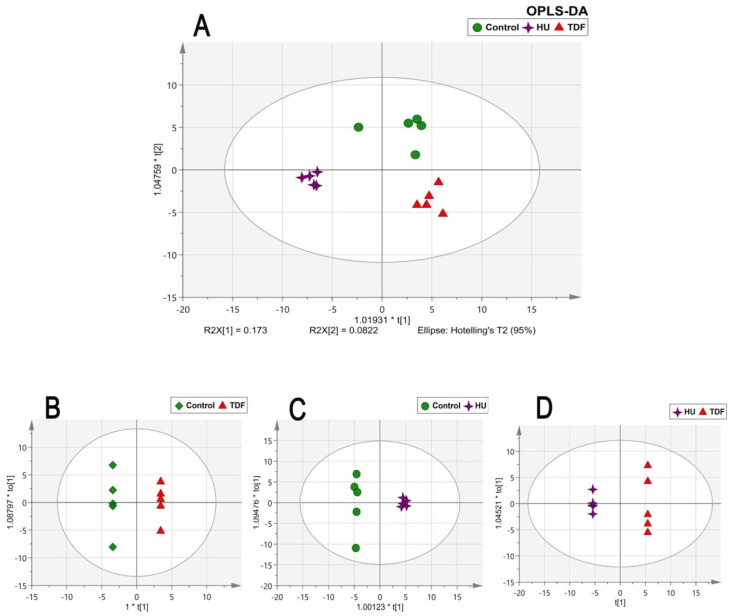
OPLS-DA statistical models for different groups. (**A**) Model with all three groups: green circles = untreated control, red triangles = β-YAC transgenic mice treated with TDF, and purple four-pointed star = β-YAC transgenic mice treated with HU. (**B**) Model comparing untreated control and treated mice with TDF for β-thalassemia. (**C**) Model comparing untreated control and treated mice with HU for β-thalassemia. (**D**) Model comparing treated mice with TDF and HU for β-thalassemia.

**Figure 4:**
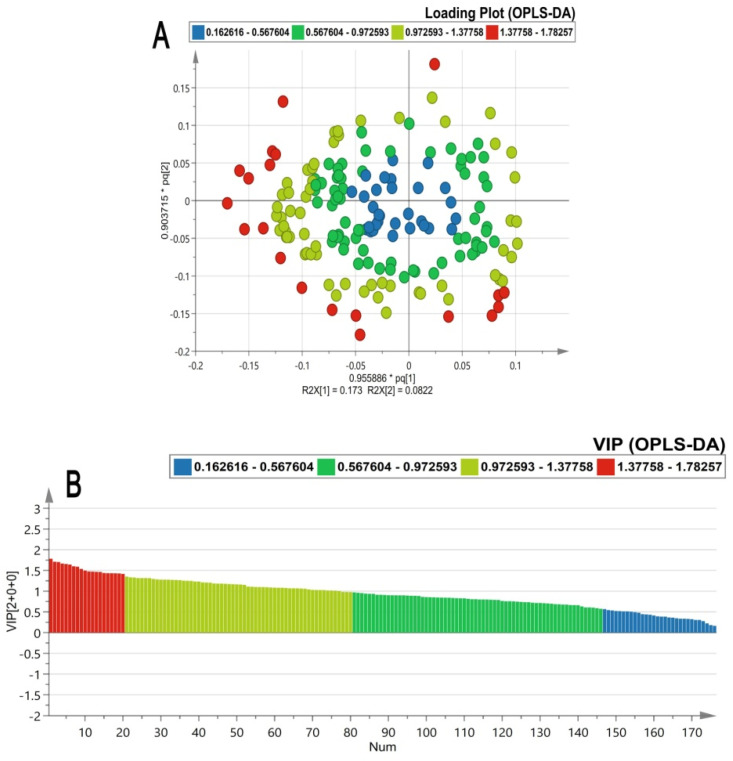
Discriminant serum metabolite extraction by (**A**) loading and (**B**) VIP plot (OPLS-DA) having VIP >1.3.

Many univariate and multivariate analyses were carried out to examine the data. MPP software was used for the univariate statistical analysis. The fold change was performed between TDF vs. control and HU vs. control groups having log FC > 1.0. Apart from univariate analysis, SIMCA software was used for the multivariate statistical analysis. The PCA score plot showed good separation between the groups based on treatment category as shown in Figure 2A and no outliers were detected as all the samples were lying within the Hotelling’s T2 region as shown in Figure 2B. Furthermore, the stability of the instrument and quality and reliability of the data were examined by the PCA score plot with QC samples as shown in Figure 2C, showing all QC lying between the samples. The OPLS-DA multivariate models Figure 3A–D disclose apparent segregation between the untreated control group, HU, and TDF groups. The discriminant metabolites were extracted by the loading (Figure 4A) and VIP plot (Figure 4B) (VIP > 1.3) of OPLS-DA;and the confidence intervals for the VIP values were found to be 95%.

The validation of the data was conducted internally with the help of ROC curve plotted for OPLS-DA model between the false positive rate (FPR, 1-specificity) and true positive rate (TPR, sensitivity). The ROC curve provides a spectrum of performance assessments and the area under the ROC (AUROC) is commonly used as diagnostic statistics of models. The AUROC values range from 1 (perfect discrimination between classes) and 0 (0.5 and lower usually means no discrimination at all). The data shows 100% sensitivity, and specificity and area under curve for untreated control, treated with TDF, and treated with HU were found to be 1, as shown in Supplementary Figure S1. All the samples were classified accurately in their own category as mentioned in the misclassification table for OPLS-DA Supplementary Table S1. 

The resulting identified metabolites underwent an ordinary one-way ANOVA (nonparametric) test to calculate *p*-values between the groups using GraphPad Prism Software. The post hoc Bonferroni statistical hypothesis testing and no matching or pairing for experimental design were selected to calculate the *p*-value by comparing the mean of each column with the mean of every column. The trends of the normalized concentration for the altered metabolites have been reported as a box plot showing significant *p*-values between the groups.

### 2.3. Discriminant Metabolites in β-YAC Transgenic Mice Induced by TDF

The serum metabolites in β-YAC transgenic mice induced by TDF show apparent alterations in their concentrations in comparison with untreated control and treatment with HU which seems to increase the HbF production. A group of metabolites L-galactopyranose, 3,3,5-trimethyldecane, 8-bromoocta-1,5-dien-3-ol, 2-methyl-6-hepten-1,2-diol, were found considerably up-regulated in TDF vs. C while not showing any significant fold change difference in HU vs. C. 5-Hydroxy-6-(1-hydroxyethyl)-2,7-dimethoxy-1,4-naphthoquinone and 2-(1-phenylethyl) phenol were up-regulated in TDF inducer but were down-regulated than the HU inducer in comparison with untreated control. Whereas L-fucose, geraniol, N-ethylcitraconimide, citraconimide, and 9.37 (unidentified) showed a decrease in level in TDF vs. C and HU vs. C, while dihexyl sulfide showed equal down-regulation in both inducers as compared to the untreated control. Some metabolites showed up-regulation in HU inducer in comparison with untreated control, however, does not show significant up/down-regulation with TDF inducer such as diacetone alcohol, myo-inositol, tritriacontane, 4-ethoxy-benzoic acid, 2-isopropyl-5-methyl-1-heptanol, while hexadecanol and arabinofuranose were down-regulated in TDF vs. C but were unable to regulate significantly in HU vs. C.

### 2.4. Pathway Analysis

**Figure 5:**
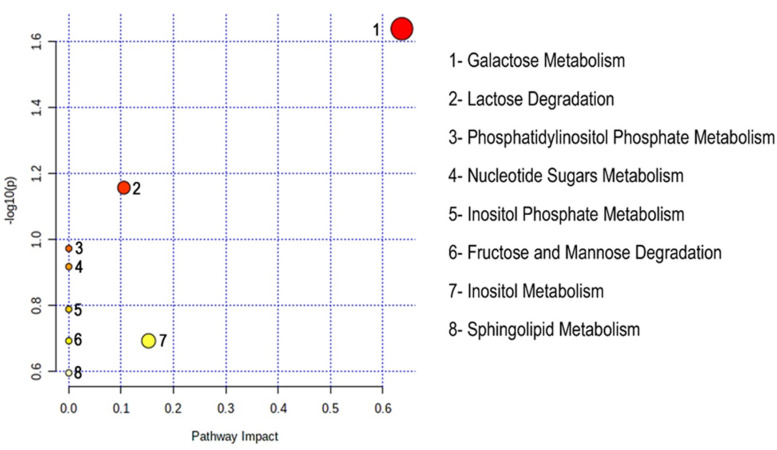
Summary of altered biological pathways in the serum samples affected by β-thalassemia in β-YAC transgenic mice resulting from MetaboAnalyst 5.0 analysis.

For the identification of metabolic biological pathways that are altered by the fetal hemoglobin-induced drugs, we used MetaboAnalyst 5.0 online software (www.metaboanalyst.ca/ (accessed on 2 February 2022)). On the basis of various databases available online such as KEGG (Kyoto Encyclopedia of Genes and Genomes), HMDB (Human Metabolome Database), and SMPDB (Small Molecule Pathway Database), a pathway analysis overview was generated by hypergeometric test, relative-betweenness centrality, and “mus musculus smpdb” parameters to show significant alterations in topology analysis as shown in Figure 5. Total 08 pathways were generated by the identified metabolite list in our analysis by MetPA according to the impact and *p*-values, shown in Table 2, named galactose metabolism, lactose degradation, phosphatidylinositol phosphate metabolism, nucleotide sugars metabolism, inositol phosphate metabolism, fructose and mannose metabolism, inositol metabolism, and sphingolipid metabolism. The metabolites which revealed disturbance among these pathways were L-fucose, L-galactose, and myo-inositol. However, we figured out only 3 out of 8 pathways with impact value > 0 that are with galactose metabolism, lactose degradation, and inositol metabolism.

## 3. Discussion

**Figure 6:**
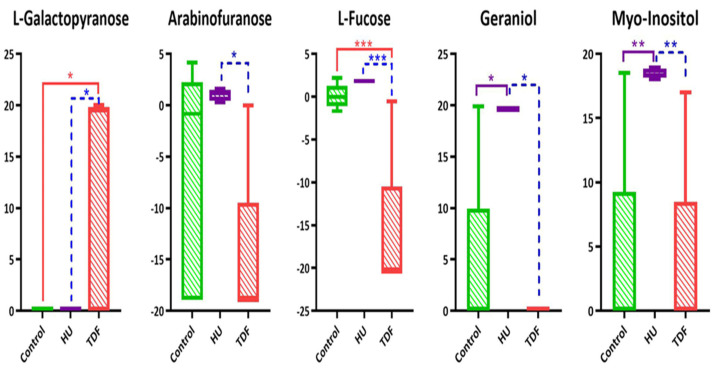
Box plots of the altered metabolites between the studied groups showing average concentrations of the specific metabolites and *p*-value calculated by one-way ANOVA (non-parametric), post hoc Bonferroni statistical hypothesis testing (* show *p*-value < 0.05, ** show *p*-value < 0.01, and *** show *p*-value < 0.001).

Several metabolites were found to be altered in the serum of β-thalassemia transgenic mice induced by TDF in comparison with treated mice induced by HU and untreated control. Among 21 metabolites, 04 are sugars. More importantly, pathway analysis also hit carbohydrate metabolites that affected the dysregulation in 03 carbohydrate metabolic pathways that correlate with the fetal hemoglobin of β-thalassemia transgenic mice. Although they did not show any regular pattern when compared with the untreated control and HU inducers. L-galactopyranose showed up-regulation whereas arabinofuranose showed down-regulation in TDF inducer. Myo-inositol and L-fucose have up-regulated in HU inducer while only L-fucose showed down-regulation in TDF inducer, and myo-inositol does not show any significant fold change in TDF inducer as shown in Figure 6. The carbohydrate metabolism can supply essential intermediates and precursors required for the production of energy by the TCA cycle and the contents tend to increase which supplies living cells with constant energy to maintain cellular homeostasis [14]. Many variables and nutrients such as metals are controlled by a homeostatic mechanism to maintain life, if not, then as a result, various diseases can happen to the individuals. Due to inborn error of metabolism or inherited defect, any homeostatic nutrient can collapse which does not threaten life immediately but can lead to serious disease by increasing oxidative stress that can be fatal as well. The increased level of L-galactopyranose and decreased level of arabinofuranose suggested direct or indirect interference with this process.

Fucose, also known as 6-deoxy-L-galactose, is a monosaccharide that occurs in glycolipids and glycoproteins and can be found in two forms, i.e., D-fucose and L-fucose. L-fucose occurs in species extending from bacteria to mammals and is a significant component of their glycoconjugates complexes where it takes part in inflammation, immunity, and metastasis of malignant cells. It is present in very low concentrations in the serum of mammals. In humans, it is one of the few sugars that exist in the L-form [15,16,17]. L-fucose takes part in the fructose and mannose metabolism pathway (sugar metabolism) where it forms fuculose with the help of L-fucose isomerase. The level of L-fucose was increased in major thalassemia patients as compared to the minor thalassemia and control ones as already reported, especially in O-blood groups as compared to the A, B, and AB type blood groups which suggested severity and complexity in the growth of the disease [18,19]. Our results illustrated down-regulation of L-fucose, as shown in Figure 6, in the β-YAC transgenic mice induced by TDF, though there was a slightly increased level in the samples induced with HU drug, suggesting TDF as a better HbF inducer for enhancing γ-globin gene activation. Another sugar, myo-inositol, has been successfully seen to defeat cancer and metabolic disorders [20] such as gestational diabetes mellitus (GDM), polycystic ovary syndrome (PCOS), thyroid, and infertility disorders [21] for the last two decades according to the scientific literature. The major thalassemic patients survived by blood transfusion for the long term, due to which they have to fight with an excess of iron causing oxidative stress (the iron overload is highly toxic and leads to organ deterioration). To remove the excess of iron, iron chelation therapy is used and one of the studies proposed inositol hexakisphosphate as an anti-oxidant that can reduce the oxidative stress of iron overload and improve immunity [22]. However, our study suggested the increased level of inositol by HU whereas no significant change has been observed by TDF.

Apart from the sugars, monoterpenes also play a significant role in the γ-globin gene expression and HbF induction [23]. Geraniol is a monoterpenoid, found in various aromatic plants as essential oils. Geraniol exhibit many biological functions including storage, cell signaling, and controls various biological processes such as apoptosis, cell survival, autophagy, cell cycle, proliferation, and metabolism. One of the studies reported that geraniol owns anti-microbial, anti-inflammatory, anti-cancer, anti-oxidant, cardio-protective, neuroprotective, and hepatoprotective effects [24], so accordingly, it might be possible that a high level of geraniol is due to oxidative stress and it lower RBCs’ membrane integrity in β–thalassemia [25]. Our study observed low levels of geraniol in the samples induced by TDF as compared to HU and control, as shown in Figure 6, resulting in TDF as a good therapeutic approach for thalassemia disease. Geraniol also plays an important role in many cancer treatments such as hepatic, colon, breast, and lung cancer [26,27,28,29] but the molecular mechanism is still basically unknown. Hence, more efforts are essential to gather further knowledge on the potency of geraniol.

We can hypothesize that the oxidative stress that occurs in β-thalassemia can be reduced by using TDF drug as a fetal hemoglobin inducer and the metabolic variations that occurred by TDF are quite different from HU which improves the pathophysiological mechanism of β-thalassemia transgenic mice.

## 4. Materials and Methods

### 4.1. Chemicals and Reagents

Active pharmaceutical ingredient (API) of Tenofovir disoproxil fumarate (TDF) (MW: 635.515) with purity >95% was obtained from the Drug Bank of Dr. Panjwani Center for Molecular Medicine and Drug Research, ICCBS, University of Karachi.

Analytical grade solvents were used for GC-MS analysis. Solvents and reagents included pyridine (Lab-Scan, Bangkok, Thailand) and MSTFA (N-methyl-N (trimethylsilyl) trifluoroacetamide) (Acros Organic, New Jersey, NJ, USA). Methanol and methoxyamine hydrochloride were purchased from Sigma Aldrich (St. Louis, MO, USA). Throughout the study, deionized water (Milli-Q) 18.2 MΩ cm, obtained from Millipore assembly (Billerica, MA, USA) was utilized.

### 4.2. Animal Study

All animal experiments were conducted in accordance with the Guide for the Care and Use of Laboratory Animals published in 1986 by the Institute of Laboratory Animal Resources of the U.S. Department of Health and Human Services [30]. The study has been approved from The Institutional Animal Care and Use Committee (IACUC) of International Center for Chemical and Biological Sciences (ICCBS) with Animal Study Protocol number 2021-017. After confirming the DNA genotyping, the β-YAC transgenic mice model was utilized, which consists of the full-length 82 kb of the human β-globin gene locus together with the locus control region (LCR) and neighboring region. Sequences of five functioning human globin genes, 5′-ε-Gγ-Aγ-δ-β-3′, are present in this region and undergo typical developmental regulation with the γ-globin gene, which was silenced shortly after birth [31]. To deliver TDF to 5- to 6-month-old β-YAC transgenic mice, intraperitoneal (i.p.) injections were used. For four weeks, TDF was dissolved in physiological saline (200 mg/kg/day) and HU (200 mg/kg/day) 6 days per week. We put our 15 mice into 3 groups, each with 5 TDF-treated, 5 HU-treated, and 5 untreated saline water control mice.

### 4.3. Sample Collection and Biological Processing

At week four after treatment, all mice were euthanized by cervical dislocation after receiving anesthetics of ketamine 70 mg/kg + xylazine 10 mg/kg. Through cardiac puncture, nearly 1 mL of blood was collected in airtight vacutainer tubes carrying EDTA for differential metabolomic expression in β-YAC transgenic mice. As previously mentioned, the γ-globin gene was expressed and HbF was produced [10]. Blood was collected, processed, and stored according to standard protocol. After coagulation, blood samples were held at room temperature before being centrifuged at 4000 rpm with the machine pre-set at 4 °C for 8 min. The supernatant was aliquoted into 200 mL safe-lock tubes and kept at −80 °C until it was processed.

### 4.4. Sample Preparation and Derivatization

The serum sample of β-YAC mice was prepared as per protocol already reported with some modifications [13]. First of all, the frozen samples were thawed on crushed ice and 60 μL of serum sample containing 16 μL of internal standard (myristic acid 1.0 mg/mL) was precipitated with 800 μL of chilled methanol and left on crushed ice for 30 min and centrifuged for 10 min on 12,000 rpm. On the other hand, 96-well plate (Strata C18-E, 55 μm pore size, 70 Å particle, 100 mg sorbent/1 mL Phenomenex, Torrance, CA, USA) was activated with 2 × 300 μL of methanol and 2 × 300 μL of milli-Q. The sample supernatant was loaded on an activated plate under vacuum (AHC-7502, Phenomenex, Torrance, CA, USA). After washing the solid phase of a plate with 2 × 300 μL of milli-Q, all the metabolites were eluted with 600 μL of methanol and collected in 96-well collection plate. The eluent was dried completely in a vacuum concentrator at room temperature and stored at −20 °C till further analysis. The next step is the derivatization of the dried samples that was executed by the addition of 30 μL of methoxylmine hydrochloride (20 mg/mL in pyridine), vortexed, and kept on thermomixer for 17 h at 350 rpm and 37 °C. All electropositive hydrogen present in the sample were converted into the volatile trimethylsilyl derivative by addition of 30 μL of MSTFA and mixed for 1 h at 65 °C. Then it was centrifuged at 12,000 rpm for 10 min and the supernatant was analyzed on GC-MS.

For robust quality assurance, quality control (QC) was also prepared by mixing 10 μL of each serum sample followed by the same preparation and derivatization method. It was subjected to batch analysis after every 05 samples.

### 4.5. GC-MS Analysis and Data Processing

GC–MS analysis was carried out on 7890A gas chromatography (Agilent Technologies, Santa Clara, CA, USA), fitted with GC autosampler 120 (PAL-LHX-AG12, Agilent Technology), coupled with a 7000 triple Quad system (Agilent Technologies, USA). ZB-5MS 30 m × 0.25 mm diameter capillary column with fused silica (Phenomenex, Torrance, CA, USA) was used, chemically bonded with a 95% dimethylpolysiloxane and 5% diphenyl cross-linked stationary phase (0.25 μm film thickness); 3.5 μL of derivatized sample was injected for GC-MS analysis. The derivatized serum sample injection was in split mode with a 10:1 split ratio and 11.2 mL/min split flow using helium as a carrier gas with 1 mL/min flow rate. First, the temperature of the oven was kept isothermal at 60 °C for 2 min, then it was raised on two ramps. In the first ramp, the temperature was raised by 5 °C/min to 240 °C for 5 min and in the second ramp, 10 °C /min to 300 °C for 4 min. The total run time for each sample was 53 min. A blank was used before, in between, and after the samples to avoid contamination. QC samples were run after every 5 samples for robust quality assurance and to check the stability of the instrument. The electron ionization (EI) source of 70 eV was used for GC-MS analysis. The acquisition of data was performed in full scan mode from 50 to 650 m/z with a 0.5 s scan time. Perfluorotributylamine (PFTBA) was used for mass calibration.

Data processing was performed by Agilent Mass Hunter Qualitative Analysis (version B.04.00). Peak integration parameters on Mass Hunter were set as previously reported [25]. The mass spectra of GC-MS peaks were compared with the existing NIST mass spectral (Wiley registry) library. For metabolite identification, ≥70% similarity index was set for matching of the spectral peaks. For further processing, the GC-MS spectral data was then exported to Mass Profile Professional (MPP) after converting it to CEF format. The data filtering includes 5000 counts for minimum absolute abundance and 03 minimum number of ions. For alignment parameters, 0.05 min RT tolerance, 0.3 match factor, and 0.2 delta MZ (low resolution) were selected. For normalization of data, external scalar was set on 1 value. The baseline to median of all samples was selected to treat all the compounds equally despite their intensities. A total of 1345 features were generated after data alignment and the data were filtered by fold change for further statistical significance analysis.

### 4.6. Statistical Analysis

The univariate analysis was performed for obtaining the features having log FC > 1.0 by Mass Profiler Professional (MPP version 12.5). The multivariate statistical tools were applied by import of the data on SIMCA^®^ 14.0, (Umetrics, Umeå, Sweden) for the detection of an outlier by Hotelling’s T2 plot, and group discrimination by principal component analysis (PCA) and orthogonal partial least square discriminant analysis (OPLS-DA) using log transform and Pareto scaling. The ROC curve was carried out to validate the model. Finally, the statistical significances were evaluated by GraphPad Prism Software (7.04 version, Inc., San Diego, CA, USA) using one-way ANOVA with a post hoc Bonferroni test and *p* < 0.05 was considered significant.

## 5. Conclusions

This study focuses on generating serum metabolic profiling of β-YAC transgenic mice treated with TDF and HU in comparison with untreated control saline group to distinguish the efficacy of both drugs on β-thalassemic mice. Our results illustrated that up/down-regulation of metabolites involved in carbohydrate metabolism will play a significant role in the pathogenesis of the disease. Furthermore, it is suggested that the decreased concentration of L-fucose and geraniol in transgenic mice treated with TDF helps in recovering towards normal, decreasing oxidative stress even much better than the transgenic mice treated with HU. Additionally, this is the first preliminary study to identify the plausible metabolites that alter the metabolism in β-thalassemia γ-globin gene, and we suggested that TDF increases the HbF production with improved efficacy in β-YAC transgenic mice.

## Figures and Tables

**Table 1:** List of identified metabolites significantly different in groups: Group 1, TDF vs. C; Group 2, HU vs. C showing up/down-regulation with log FC > 1 and VIP > 1.3.

S.No.	Name	Molar Mass	RT	VIP Value	log (FC)		Regulation		*p*-Value		
					TDF vs. C	HU vs. C	TDF vs. C	HU vs. C	TDF vs. C	HU vs. C	TDF vs. HU
**1**	Geraniol	154.25	25.05	1.7825	−3.97	15.69	↓	↑	ns	*	*
**2**	L-Fucose	164.16	30.83	1.7098	−16.51	1.75	↓	↑	***	ns	***
**3**	Diacetone alcohol	116.16	11.04	1.6653	−0.73	2.10		↑	ns	ns	ns
**4**	Myo-Inositol	180.16	32.76	1.6540	−0.30	14.78		↑	ns	**	**
**5**	N-Ethylcitraconimide	139.15	9.30	1.3157	−3.65	13.43	↓	↑	ns	*	**
**6**	Citraconimide	111.10	11.49	1.2955	−3.37	12.82	↓	↑	ns	*	**
**7**	Dihexyl sulfide	202.40	18.35	1.6420	−4.00	−4.00	↓	↓	*	*	ns
**8**	Tritriacontane	464.90	34.56	1.6000	−0.80	1.75		↑	ns	*	***
**9**	2-Methyl-6-hepten-1,2-diol	144.21	9.51	1.5899	4.00	0	↑		*	ns	*
**10**	3,3,5-Trimethyldecane	184.36	19.16	1.5363	4.00	0	↑		*	ns	*
**11**	5-Hydroxy-6-(1-hydroxyethyl)-2,7-dimethoxy-1,4-naphthoquinone	278.26	21.33	1.4968	10.10	15.43	↑	↑	ns	**	ns
**12**	Nonacosane	408.80	38.39	1.4755	0.42	0.89			ns	ns	ns
**13**	4-ethoxy-Benzoic acid	166.17	21.11	1.4720	−0.11	1.00		↑	ns	ns	*
**14**	Eicosane	282.50	33.23	1.4655	0.09	0.79			ns	ns	ns
**15**	Hexadecanol	242.44	29.28	1.4639	−4.00	0.63	↓		ns	ns	*
**16**	L-Galactopyranose	180.16	30.83	1.4379	4.00	0	↑		*	ns	*
**17**	2-Isopropyl-5-methyl-1-heptanol	172.31	16.28	1.4338	0.17	1.39		↑	ns	ns	ns
**18**	2-(1-phenylethyl)Phenol	198.26	23.40	1.4331	2.04	2.95	↑	↑	ns	ns	ns
**19**	8-Bromoocta-1,5-dien-3-ol	205.09	48.03	1.4309	4.00	0	↑		*	ns	*
**20**	Arabinofuranose	150.13	26.86	1.4164	−1.24	−0.22	↓		ns	ns	*
**21**	Unidentified	--	9.37	1.4271	−4.00	1.54	↓	↑	ns	ns	*

TDF = Tenofovir disoproxil fumarate; HU = Hydroxyurea; C = Untreated control; ↑ = up regulation; ↓ = down regulation; * = *p*-value < 0.05; ** = *p*-value < 0.01; *** = *p*-value < 0.001; ns = non-significant.

**Table 2:** List of altered metabolic pathways for β-thalassemia in comparison with untreated control and treated mice.

Pathways	Total	Hits	Hits Name	Raw *p*	FDR	Impact
Galactose Metabolism	31	2	L-Galactose, Myo-inositol	0.022974	1	0.63665
Lactose Degradation	9	1	L-Galactose	0.069671	1	0.10526
Phosphatidylinositol Phosphate Metabolism	14	1	Myo-inositol	0.10651	1	0
Nucleotide Sugars Metabolism	16	1	L-Galactose	0.12089	1	0
Inositol Phosphate Metabolism	22	1	Myo-inositol	0.1628	1	0
Fructose and Mannose Degradation	28	1	Fucose	0.20296	1	0
Inositol Metabolism	28	1	Myo-inositol	0.20296	1	0.15251
Sphingolipid Metabolism	36	1	L-Galactose	0.25388	1	0

## Data Availability

Not applicable.

## Supplementary Materials

The supporting information can be downloaded at: https://www.mdpi.com/article/10.3390/ijms232415750/s1.
