# Supplementary material for: Metabolomics Study of Serum Samples of β-YAC Transgenic Mice Treated with Tenofovir Disoproxil Fumarate
Source: Int J Mol Sci. 2022 Dec 12;23(24):15750. doi: 10.3390/ijms232415750 (PMC9778960; doi:10.3390/ijms232415750)
Supplement: Supplementary file 1 [file ijms-23-15750-s001.zip › ijms-1879028-supplementary.pdf]

Supplementary Material

Metabolomics study of serum samples of β-YAC transgenic mice treated with tenofovir disoproxil fumarate

Sindhia Kumari[1], Faisal Khan[2], Amna Jabbar Siddiqui[2], Nurmeen Adil[1], Jalal Uddin[3], Mufarreh Asmari[3] and Syed Ghulam Musharraf[1,2,4*]

[1] *H.E.J. Research Institute of Chemistry, International Center for Chemical and Biological Sciences, University of Karachi, Karachi-75270, Pakistan*

[2] *Dr. Panjwani Center for Molecular Medicine and Drug Research, International Center for Chemical and Biological Sciences, University of Karachi, Karachi-75270, Pakistan*

[3]*Department of Pharmaceutical Chemistry, College of Pharmacy, Abha 62529, Saudi Arabia*

[4] *The Affiliated T.C.M Hospital of Southwest Medical University, Luzhou 646099, Sichuan, China*

*Corresponding author

Correspondence:

Prof. Dr. Syed Ghulam Musharraf, Ph.D.

H. E. J. Research Institute of Chemistry, International Center for Chemical and Biological Sciences, University of Karachi.

Mailing address: Lab: no: 308, H. E. J. Research Institute of Chemistry, International Center for Chemical and Biological Sciences, University of Karachi, Karachi, Pakistan

Tel.: +92 321 8270867

Telefax: (+ 92-21) 4819018, 4819019.

E-mail address: musharraf1977@yahoo.com; musharraf@iccs.edu

† Electronic supplementary information (ESI) available

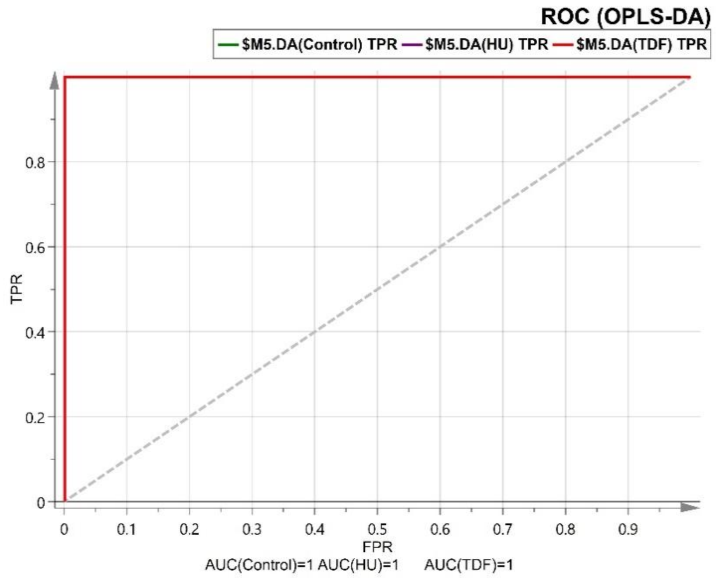

Supplementary Figure S1. ROC (Receiver Operating Characteristics) curve for the internal validation of OPLS-DA model with 100% specificity on x-axis and sensitivity on y-axis showing area under curve value 1.

Supplementary Table S1. Misclassification table for generated OPLS-DA model.

	**Members**	**Correct**	**Control**	**HU**	**TDF**	**No class (YPred <= 0)**
**Control**	5	100%	5	0	0	0
**HU**	5	100%	0	5	0	0
**TDF**	5	100%	0	0	5	0
**No class**	0		0	0	0	0
**Total**	15	100%	5	5	5	0
**Fisher's prob.**	7.90E-06					
